# Comparison of Visual Outcomes and Quality of Life in Patients with High Myopic Cataract after Implantation of AT LISA Tri 839MP and LS-313 MF30 Intraocular Lenses

**DOI:** 10.1155/2022/5645752

**Published:** 2022-02-28

**Authors:** Jiying Shen, Limei Zhang, Shuang Ni, Lei Cai, Haike Guo, Jin Yang

**Affiliations:** ^1^Department of Ophthalmology, Shanghai Heping Eye Hospital, Shanghai, China; ^2^Department of Ophthalmology and the Eye Institute, Eye and Ear Nose and Throat Hospital, Fudan University, Shanghai, China; ^3^The Key Laboratory of Myopia, Ministry of Health, Shanghai, China; ^4^Shanghai Key Laboratory of Visual Impairment and Restoration, Shanghai, China; ^5^Key National Health Committee of the Key Laboratory of Myopia, Fudan University, Shanghai, China; ^6^The Key Laboratory of Myopia, Chinese Academy of Medical Sciences, Shanghai, China

## Abstract

**Purpose:**

To investigate the benefits of multifocal lens in patients with high myopic cataract and compare the clinical effects between AT LISA tri 839MP and MPlus LS-313 MF30 intraocular lenses (IOLs) in high myopic eyes.

**Methods:**

This retrospective cohort study analyzed 60 eyes with axial length >26 mm in 40 patients. Thirty eyes were implanted with MF30, and the remaining 30 eyes were implanted with 839MP. Postoperative uncorrected distance visual acuity (UDVA), best corrected distance visual acuity (BCDVA), uncorrected intermediate visual acuity (UIVA) and uncorrected near visual acuity (UNVA), defocus curve, modulation transfer function (MTF) curve, Strehl ratio (SR), and complications were compared between the two groups.

**Results:**

All vision outcomes were significantly improved in both groups (*p* < 0.05). There was no significant between-group difference in UDVA at 1 and 3 months postoperatively (*p* > 0.05). However, UIVA and UNVA were significantly better in the 839MP group (*p* < 0.05). The VF-14 score, especially for near vision quality, was significantly higher in the MF30 group (2.2 ± 0.9 vs. 0.8 ± 0.7; *p* ≤ 0.001). The SR of both groups significantly increased postoperatively (*p* < 0.05). All the 3-month MTF curve values (MTF 10 total, MTF 10 internal, MTF 30 total, and MTF 30 internal) were significantly better in the 839MP group (*p* < 0.05). Meanwhile, all the high-order aberration values (coma, spherical aberration, and trefoil) were significantly greater in the MF30 group (*p* < 0.05).

**Conclusion:**

Multifocal IOL implantation achieves good quality of distance, intermediate, and near vision in patients with high myopia, improving their quality of life. Both 839MP and MF30 IOLs can provide good distance vision, but 839MP performs better in near and intermediate vision. However, for some patients with an extra-long optic axis, MF30 may be a good choice because of its wider range of degrees.

## 1. Introduction

The incidence of high myopia is increasing worldwide, with the number of patients with high myopia and complicated cataracts markedly increasing [1]. Patients expect spectacle-free vision after cataract surgery. Surgery for highly myopic eyes is challenging. The most common surgical strategy is implanting monofocal intraocular lens (IOL) and leaving −2.5 D to −3.0 D myopia. However, although this achieves excellent near vision, it is also associated with loss of regulation and stereoscopic vision in the active state after surgery. The development of cataract phacoemulsification and advances in surgical technology and IOL calculation methods have greatly improved the predictability of refractive results after cataract surgery for high myopia. Although still controversial, an increasing number of multifocal IOLs (MIOLs) have been used in highly myopic eyes. Some studies [2–4] have also reported full range of vision after MIOL implantation in many patients with high myopia, significantly improving the patients' quality of life (QOL). Given their multiple focus, these IOLs provide good vision for activities at multiple distances [5–7]. However, they are also complicated by undesirable effects such as glare, halos, and reduced contrast sensitivity.

At present, only a limited type of MIOLs, including AT LISA tri 839MP (Carl Zeiss Meditec AG, Jena, Germany) and MPlus LS-313 MF30 (Oculentis, Holland), can be used in high myopia due to the limitation of the degree range. Both 839MP and MF30 have been reported to achieve good outcomes in patients with high myopia [8,9]. However, few studies have compared the visual quality between these two different lenses in patients with high myopic cataract. As such, this study aimed to compare the clinical benefits between AT LISA tri 839MP and MPlus LS-313 MF30 IOLs in high myopic cataract. Toward this goal, we evaluated the postoperative visual quality and compared the feasibility of these two IOLs in patients with high myopia.

## 2. Methods

### 2.1. Study Design and Participants

This was a retrospective cohort study of 60 eyes from 40 patients diagnosed with cataract and high myopia who underwent phacoemulsification cataract extraction combined with MIOL implantation at Shanghai Heping Eye Hospital, Shanghai, China, between September 2018 and July 2021. The inclusion criteria were as follows: age >18 years, length of optic axis >26 mm, irregular corneal astigmatism <0.3 um; postoperative corneal astigmatism ≤0.75 D, clear intraocular media, available to comply with examination procedures, and written informed consent for participation in the study. The exclusion criteria were pupil centroid shift; pupil size >5 mm or <2 mm in dim light; amblyopia; previous ocular surgery; ocular pathologies such as diabetic retinopathy, macular degeneration, and glaucoma with field defects; lifestyle; and work-related factors, such as pilots, professional drivers, and architects.

Among the 40 patients, there were 3 patients with one eye implanted in MF30 and another eye in 839MP; they simultaneously belonged to both groups, so there were totally 23 patients (30 eyes) and 20 patients (30 eyes) who underwent regional refraction MIOL (MF30) implantation and diffraction MIOL (839MP) implantation categorized to the MF30 group and 839MP group, respectively. Patients in the two groups were enrolled under the same conditions. All patients were followed up for 3 months. The characteristics of the lenses are listed in Table 1.

### 2.2. Preoperative Examination

Preoperative examination included (1) uncorrected distance visual acuity (UDVA); (2) best corrected distance visual acuity (BCDVA); (3) uncorrected near visual acuity (UNVA); (4) subjective refraction; (5) corneal topography assessed with Pentacam Comprehensive Eye Scanner (Oculus Optikgeraete GmbH; Wetzlar, Germany); (6) slit-lamp biomicroscopy of the anterior and posterior segments with a Volk lens, optical coherence tomography, scanning laser ophthalmoscopy, retinal fiber nerve layer, Pascal tonometry, and biometry (IOL-Master 700; Carl Zeiss Meditec AG); (7) higher-order aberrations (HOA); and (8) Strehl ratio (SR).

### 2.3. Surgical Technique

The surgery was performed by the same senior physician, and standard phacoemulsification was used for cataract extraction. In all patients, topicamide was used to fully dilate the pupil, and cocaine eye drops were used for surface anesthesia. A 2.2 mm transparent corneal incision was made at 130°, and a central continuous circular capsulorhexis was performed with a diameter of 5.5 mm. After water separation and stratification, phacoemulsification was performed to extract the lens nucleus, and the I/A system was used to extract the lens cortex. An IOL was implanted after the viscoelastic agent was injected into the anterior chamber and pouch. The I/A system was used to remove the viscoelastic agent, and the incision was watertight. The IOL power was calculated using optical biometry (IOL-Master 700; Carl Zeiss Meditec, Jena, Germany) and Barrett formulas. The target refraction was 0 in the operative eye with an axis between 26 mm and 30 mm. Meanwhile, considering that the ultralong eye axis is prone to farsighted drift, the target diopter was kept within −0.5 D in the surgical eyes with an axis >30 mm.

### 2.4. Postoperative Follow-Up and Assessments

The patients were followed up at 1 week, 1 month, and 3 months postoperatively. Patient satisfaction was assessed using the modified Vision Acuity and Visual Function Index 14 (VF-14) [10] at 3 months. The VF-14 has a total of 14 questions. A score was assigned to each answer, and a higher score indicated poorer QOL. The patients were also asked questions about their satisfaction and dissatisfaction with vision and whether there was no vision disorder in daily life [11]. In addition, we recorded any side effects or complications during the 3-month period.

### 2.5. Outcome Measures

The primary outcome measure was visual acuity measured according to UDVA and BCDVA at 5 m; UIVA at 80 cm; and UNVA at 40 or 33 cm. Visual examination was performed twice under sufficient lighting, and the international standard visual acuity table was used. The secondary outcome measures were as follows: (1) HOA such as coma, spherical, trefoil, and secondary astigmatism measured using internal and total values at a 3-mm pupil size with the HOYA iTrace ray-tracing system (Tracey Technologies, Houston, TX, USA); (2) the SR was also measured using internal and total values at a 3-mm pupil size with the HOYA iTrace ray-tracing system; and (3) defocus curves for each MIOL, obtained by plotting the mean visual acuity with 11 values of defocus from +2.0 D to −3.0 D on the ETDRS chart in logMAR units. The defocus curve simulates the patient's visual acuity at different distances by placing positive and negative lenses in 0.5 D increments in front of the patient's eyes. The measurements were performed by adding lenses to BCDVA.

### 2.6. Statistical Analysis

Measurement data were expressed as *X* ± *S*. Between-group comparisons by sex were performed using the *χ*^2^ test. Age, axial length (AL), anterior chamber depth (ACD), lens thickness (LT), white-to-white (WTW), and dysfunctional lens index (DLI) were compared using the *t*-test. Repeated-measurement analysis of variance was used for between-group comparison of pre- and postoperative visual acuity, HOA, and defocus curve. All statistical analyses were performed using SPSS 17.0. Statistical significance was set at *p* < 0.005.

## 3. Results

### 3.1. Baseline Characteristics

In total, 40 patients (60 eyes) were finally included in the analysis; of them, 30 eyes belonged to the MF30 group and 30 eyes belonged to the 839MP group. There was no significant difference in age (59.8 ± 9.2 vs. 54.4 ± 12.5 years, *p*=0.065), the proportion of men (43.5% [*n* = 10] vs. 35.0% [*n* = 7], *p* > 0.05), and the proportion of women (56.5% [*n* = 13] vs. 65% [*n* = 13], *p* > 0.05) between the MF30 and 839MP groups. There were also no significant between-group differences in the preoperative UDVA and BCVA. In addition, optical biometry, such as AL, ACD, LT, and WTW, and the severity of cataract indicated by DLI were also not significantly different (Table 2).

### 3.2. Visual Outcomes

**Figure 1:**
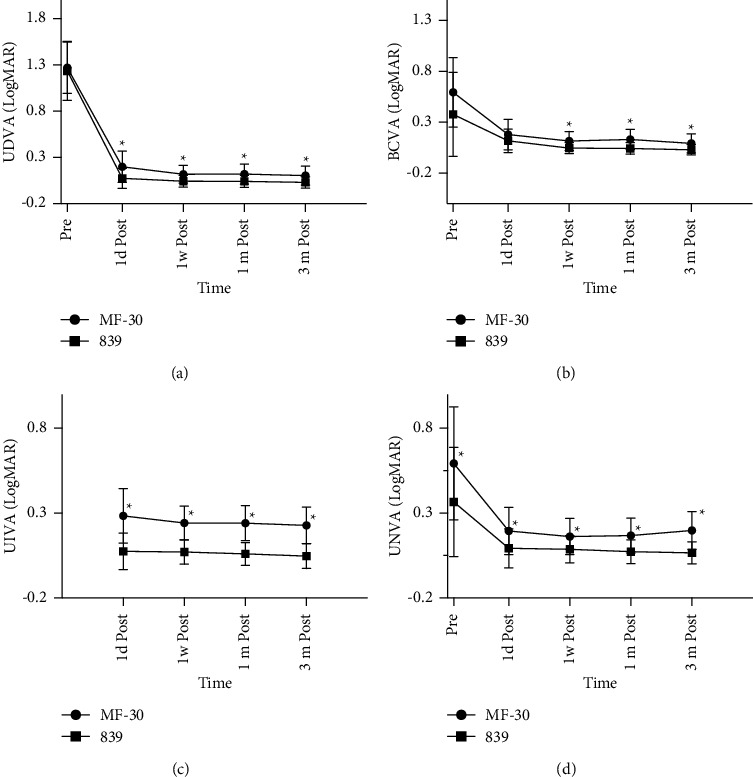
The preoperative and postoperative visual outcomes of two MIOL groups in 3 months. All data were presented as mean ± SD. (a) UDVA (logMAR). (b) BCVA (logMAR). (c) UIVA (logMAR). (d) UNVA (logMAR). ^*∗*^*p* < 0.05.

Postoperative refractive status of patients in the two groups were mostly emmetropia. The spherical equivalent measured by automatic optometry were −0.52 ± 0.48 D in the MF30 group and −0.08 ± 0.16 D in the 839MP group. Compared with the MF30 group, the 839MP group showed significantly better 3-month UDVA (0.10 ± 0.10 vs. 0.03 ± 0.07, *p* ≤ 0.001), BCDVA (0.09 ± 0.09 vs. 0.03 ± 0.05, *p*=0.002), and UNVA (0.20 ± 0.11 vs. 0.07 ± 0.07, *p* ≤ 0.001). UNVA was also significantly different between the two groups at all three visits. Similarly, the 3-month UIVA was significantly better in the 839MP group (0.23 ± 0.11 vs. 0.05 ± 0.08, *p* ≤ 0.001) (Figure 1).

### 3.3. Defocus Curve

**Figure 2:**
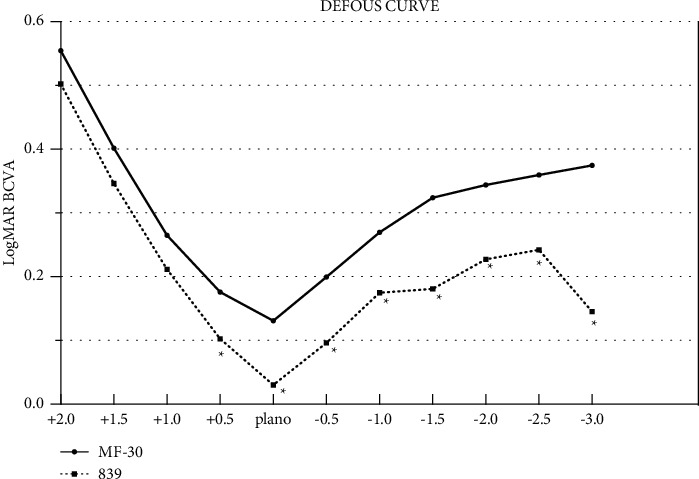
The MTF 10 and 30 (both total and internal) of two MIOL groups, included preoperative and postoperative at 3 months. A comparision of MTF 10 and 30 (both total and internal) between two groups after surgery at 3-month follow-up. All data were presented as mean ± SD. ^∗^Significant difference (*p* < 0.05).

The postoperative defocus curves of the two groups are shown in Figure 2. In the 839MP group, defocus curves showed a bimodal pattern, with the far and near focus at 5 m and 40 cm, respectively; the corresponding peaks were (0.030 ± 0.036) logMAR and (0.145 ± 0.069) logMAR, providing better visual acuity than 0.1 logMAR within +0.5 to −0.5 D and 0.2 logMAR within −1.0 to −1.5 D and −3.0 D. Meanwhile, the defocus curve in the MF30 group only showed a one-peak shape, with the focal point at 5 m. The corresponding peak value was 0.131 ± 0.099 logMAR, providing better visual acuity than 0.2 logMAR within +0.5 D to −0.5 D. Significant differences between the MF30 group and 839MP group defocus curves were detected for the following vergences: +0.5, 0.0, −0.5, −1.0, −2.0, −2.5, and −3.0 D (all *p* < 0.05) (Figure 2).

### 3.4. Quality of Life and Objective Visual Quality

**Figure 3:**
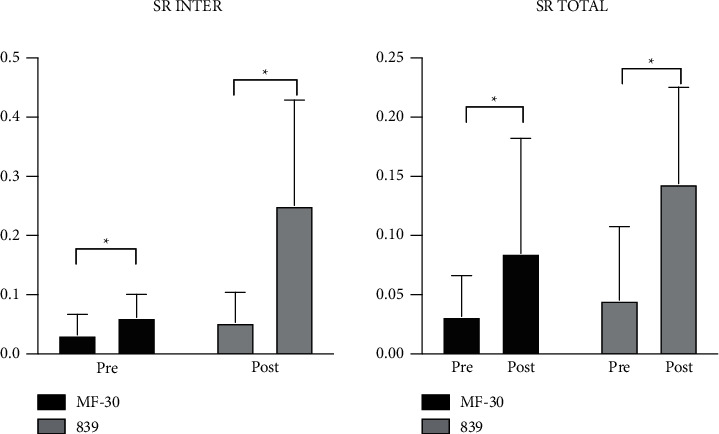
Binocular defocus curves in MF30 and 839MP groups. All data were presented as mean. ^∗^Significant difference (*p* < 0.05).

**Figure 4:**
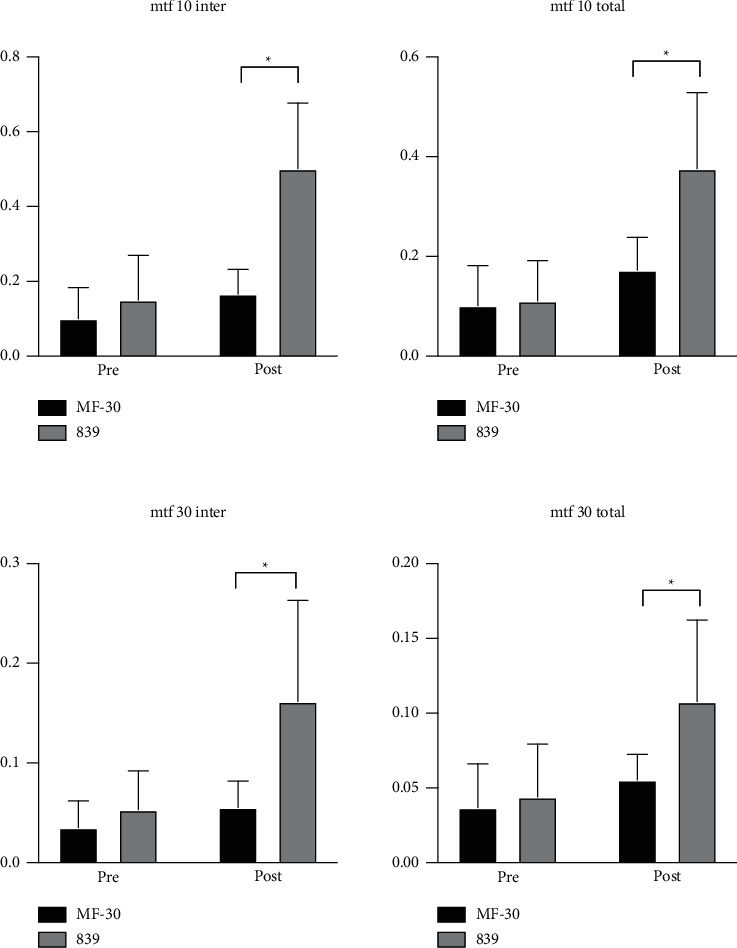
The comparision of preoperative and postoperative Strehl ratio (SR) total and internal of two MIOL groups at 3 months. The ordinate means the specific value of SR. All data were presented as mean ± SD. ^∗^Significant difference (*p* < 0.05).

All patients answered the VF-14 questionnaire. The VF-14 score was significantly higher in the MF30 group (2.2 ± 0.9 vs. 0.8 ± 0.7, *p* ≤ 0.001) (Table 2). Meanwhile, there were minimal differences in the level of satisfaction between the two groups. However, the MF30 group had significantly lower satisfaction with near vision quality. Similar results were observed for objective visual quality, such as the SR and MTF curve. Both postoperative total SR and internal SR were significantly increased compared with preoperative values of the two groups, and the difference was statistically significant. However, the range was larger in the 839MP group than in the MF30 group (Figure 3). In addition, both the 3-month MTF 10 total (0.173 ± 0.065 vs. 0.376 ± 0.152, *p* ≤ 0.001) and MTF 10 internal (0.166 ± 0.066 vs. 0.502 ± 0.175, *p* ≤ 0.001) were significantly higher in the 839MP group. Furthermore, the MF30 group showed significantly lower MTF 30 total (0.056 ± 0.017 vs. 0.108 ± 0.155, *p* ≤ 0.001) and MTF 30 internal (0.056 ± 0.026 vs. 0.162 ± 0.101, *p* ≤ 0.001) at 3 months (Figure 4).

### 3.5. High-Order Aberrations and Postoperative Complications

At 3 months postoperative, almost all the HOA values (coma, spherical aberration, trefoil) were significantly greater in the MF30 group than in the 839MP group (*p* < 0.05) (Table 3). No serious postoperative complications were noted during the 3-month follow-up in either group. However, there were three cases of posterior capsule opacification in the 839MP group, and this caused diminution of vision and needed Nd:YAG laser capsulotomy. In the MF30 group, one patient developed asymmetric constriction of the lens pouch that caused the IOL to shift upward.

## 4. Discussion

Advances in refractive cataract have led to the development of IOLs with ingenious design and different functions, such as trifocal IOLs and segmental refractive IOLs. Both 839MP and MF30 are frequently used in high myopic cataracts owing to their wide range of spherical powers. Good outcomes of MIOLs in eyes with high myopia have been reported in recent years [12–15]. However, there are few reports on the optimal IOL for patients with high myopia. Our study evaluated the postoperative visual quality between the two multifocal IOLs in high myopic cataract and compared the clinical benefits and the feasibility of these two IOLs in patients with high myopia.

The results of this study showed that 1-month UDVA was almost as similar as that at 3 months in both 839MP and MF30. The patients' far, medium, and near visual acuity and diopter reached the expected refractive correction. Furthermore, most patients had stable visual acuity 1 month after surgery, and the operation was predictable. Compared with preoperative eyesight, postoperative eyesight was improved as indicated by a significant difference between baseline and postoperative UDVA, BCDVA, UIVA, and UNVA in both groups. Similarly, the postoperative SRs were significantly increased in the two groups. This increase in SRs indicated the improvement of not only vision but also vision quality. Collectively, these results support the effectiveness of multifocal IOLs.

However, the UIVA and UNVA in the 839MP group were significantly better than those in the MF30 group. This indicates that trifocal IOLs can provide better whole-course visual acuity in patients with high myopia. Patients in the 839MP group reported good satisfaction with far, medium, and near visual acuity 3 months postoperatively, consistent with previous results [13,14,16]. This may be because the MF30 IOL has a lower attachment degree of near power (+3.00 D). High myopic eyes require more near power to reach the same level as the normal eye.

Several clinical studies have shown that after implantation of regional refraction, MIOL patients can not only obtain good near and far vision [17–19] but also have almost no limitation in middle-distance operation such as using computers [20]. In contrast, we found a different result. Analysis of defocus curves at 3 months postoperative showed a bimodal pattern in the 839MP group, with the far and near focus at 5 m and 40 cm, respectively. Meanwhile, the defocus curve in the MF30 group only showed a one-peak pattern, with a focal point at 5 m. This could be because all patients in this study had high myopic cataracts. When used in emmetropia, regional refraction IOLs can still provide a continuous vision range. The 839MP group had better vision than the MF30 group at the following vergences: +0.5, 0.0, −0.5, −1.0, −2.0, −2.5, and −3.0 D, and the difference was significant. This further confirms the result that trifocal IOLs achieve better medium and near vision and are more suitable for patients with middle-distance requirements such as those with computer work.

Objective visual quality can be quantified by the MTF curve, SR, and other indicators [21,22]. HOYA iTrace can directly collect PSF to calculate SRs and translate it into an MTF curve. The MTF curve reflects the different spatial frequencies in the clear degree of imaging. A low spatial frequency usually reflects the ability to see the object contour, while a high spatial frequency reflects the ability to distinguish fine objects. In this study, we used the MTF values under a spatial frequency of 10 to evaluate far visual acuity and MTF values under a spatial frequency of 30 to evaluate near visual acuity. The improvement in visual quality was reflected by comparing between pre- and postoperative SR values. The results showed that the total and intraocular SR values were significantly improved after surgery in both groups, and the difference between pre- and postoperative values was significant. This suggests that both IOLs can effectively improve visual quality. Intergroup comparisons showed that MTF 10 and MTF 30 in both internal and total eye groups were improved after surgery. However, the 839MP group showed significantly better improvements than the MF30 group. These results indicate that trifocal IOLs can result in more stable and excellent visual quality than segmental refractive IOLs in patients with high myopia combined with cataracts.

Similar results were observed for HOAs. Some patients had excellent postoperative vision but still complained of blurred vision, glare, and decreased night vision, and this is closely related to HOAs. At 3 months postoperatively, both coma and trefoil, as measured by the iTrace ray-tracing system, were greater in the MF30 group than in the 839MP group. Some studies have analyzed the coma of regional refraction MIOL and found that the values of both the far and near optical regions were 0. However, when measured by traditional wavefront aberration instruments, the light emitted from off-axis points is refracted through the upper and lower optical planes of the IOL, resulting in a large vertical coma [23,24]. Although this design lengthens the depth of focus and improves near vision, the instrument cannot be distinguished during measurement [25,26]. Automatic optometry cannot recognize neither, so the measurement result usually shows a myopic astigmatism error about −1.25 D. Meanwhile, the concentric diffraction ring design on the rear surface of the diffracted MIOL has lesser interference on aberration measurement and a smaller corresponding coma. Multiple clinical studies [18] have reported that coma and HOAs such as coma and trefoil in implanted regional refraction MIOL cannot adequately explain postoperative visual quality of patients. Furthermore, instrumental measurement is greatly affected by the additional fan-shaped optical area, and thus, the reference value is limited.

The VF-14 scores were consistent with the visual outcome assessment findings. Overall satisfaction was very high in both groups despite limitations in fine object recognition, such as reading newspapers and threading a needle. Near vision quality was significantly better in the 839MP group, but there was no remarkable difference in driving comfort. This might be due to good objective outcomes at distance vision in both groups. Another difference between the two groups was the difficulty in walking up and down the stairs. The VF-14 scores showed worse adaptation in the MF30 group. This might be because if IOLs are placed vertically, the lower part of the lens is attached with +3.0 D near-area. When looking down, the optical axis may enter the eye through the near area, resulting in blurred vision and reduced sense of distance and making it difficult to walk up and down the stairs. This is more common in patients with large pupils.

We also surveyed the patients about photic phenomena, such as glare and halo. The incidence and perception level of halo and glare were significantly lower in the MF30 group. One possible explanation for this finding is that unlike the AT LISA tri 839MP IOL, the MF30 IOL does not have diffractive steps. Many diffractive steps are responsible for glare and halo [27]. Patients in the MF30 group usually complained about a triangle-shaped halo while driving or using a mobile phone. However, both groups reported good driving scores, which could be explained by the fact that after 3 months of neuroadaptation, glare and halo effects were no longer perceived by the patients as detrimental for driving. However, this hypothesis requires further research in a larger sample with a longer follow-up period.

In addition, we found 3 cases of severe posterior capsular opacities within 3 months postoperatively in 839MP group, and this required YAG laser posterior capsulotomy to improve vision. Although this complication was also observed in the MF30 group, the severity was lower than that in the 839MP group. This may be related to the fact that both of these IOLs are hydrophilic acrylates. In addition, patients with posterior capsular opacities were obviously younger. Furthermore, high myopia is not an influencing factor of increased probability of occurrence [28]. Concurrently, one patient in the MF30 group had obvious anterior capsule contraction, leading to an upward shift in the effective position of the IOL. This caused the optical axis to mostly reach the eye through the near-visual area and resulted in blurred vision and decreased visual quality. We will continue to monitor other patients to determine if similar events occur.

This study has some limitations. First, the sample size was inadequate to obtain robust conclusions. Second, we could not obtain reading parameters because standardized reading charts were not available. Moreover, it is difficult to determine the incidence of photic phenomena because many IOL studies use self-made questionnaires to capture patient-related outcomes, and these questionnaires are not standardized. Third, this was not a randomized study; the patients and the surgeon were aware of the type of lens used. Despite these limitations, this study provides important data on the comparison between 839MP and MF30 for high myopia. In further study, we will expand the sample size and extend follow-up time to obtain more data.

## 5. Conclusion

MIOL achieves good distant, intermediate, and near visual quality in patients with high myopia and cataract and significantly reduces postoperative dependence on glasses, improving QOL. Furthermore, the patients did not show retinal vulnerability in this study but were still required long-term follow-up, especially routine check of the retina. In particular, both 839MP and MF30 can provide good distant vision, but 839MP has superior intermediate and near vision benefits. Meanwhile, MF30 has a wider range of degrees and may thus be the optimal choice for patients with ultralong ocular axis. Moreover, 839MP is two times more costly than MF30 and is associated with a high incidence of posterior capsule opacification in young patients. These factors should be considered when selecting the most suitable IOL.

## Figures and Tables

**Table 1:** The properties of the two IOLs used in the present study.

Name	AT LISA tri 839MP	MPlus LS-313 MF30
Optics	Diffractive	Segmental refractive
Material	Hydrophilic acrylic	Hydrophilic acrylic
Near add (D)	+3.33	+3.00
Dioptric range (D)	0.0 to +32.0	−10.0 to +35.0
Edge design	360° square edge	360° square edge
A constant	118.6	118.5
Refractive index	1.48	1.48
Optic diameter (mm)	6.0	6.0
Overall diameter (mm)	11.0	11.0

**Table 2:** The baseline characteristics and VF-14 scores of patients in both IOL groups in the study.

Characteristics	Group 1		Group 2		*p* value
	Mean	SD	Mean	SD	
Age	59.8	9.2	54.4	12.5	0.065
Axial length (mm)	28.79	2.56	27.97	1.90	0.167
Depth of anterior chamber (mm)	3.35	0.28	3.38	0.28	0.627
Lens thickness (mm)	4.44	0.32	4.32	0.33	0.158
White-to-white (mm)	11.8	0.4	11.8	0.5	0.599
VF-14 score	2.2	0.9	0.8	0.7	≤0.001
Preoperative DLI	2.63	2.24	3.60	2.60	0.127

**Table 3:** High-order aberrations of two different MIOL groups at 3 months after surgery in 60 eyes of 40 patients.

Aberrations	Group 1		Group 2		*p* value
	Mean	SD	Mean	SD	
Preoperative HOA total (*μ*)	1.734	3.914	3.157	14.827	0.613
Preoperative HOA internal (*μ*)	1.736	3.924	3.151	14.845	0.616

*Postoperative total*					
HOA (*μ*)	0.296	0.054	0.137	0.096	≤0.001
Coma (*μ*)	0.160	0.047	0.052	0.038	≤0.001
Spherical (*μ*)	0.030	0.040	0.000	0.046	≤0.001
Trefoil (*μ*)	0.173	0.062	0.096	0.109	0.011
Secondary astigmatism (*μ*)	0.039	0.021	0.033	0.042	0.002

*Postoperative internal*					
HOA (*μ*)	0.291	0.063	0.119	0.101	≤0.001
Coma (*μ*)	0.154	0.050	0.050	0.035	≤0.001
Spherical (*μ*)	0.014	0.031	−0.020	0.038	≤0.001
Trefoil (*μ*)	0.168	0.066	0.064	0.091	≤0.001
Secondary astigmatism (*μ*)	0.037	0.022	0.032	0.043	0.510

## Data Availability

The data sets generated and/or analyzed during the current study are available from the corresponding author on reasonable request and approval by the institutional ethics committee.
